# The situation of nursing education in Latin America and the Caribbean towards universal health

**DOI:** 10.1590/1518-8345.2232.2913

**Published:** 2017-05-11

**Authors:** Silvia Helena De Bortoli Cassiani, Lynda Law Wilson, Sabrina de Souza Elias Mikael, Laura Morán Peña, Rosa Amarilis Zarate Grajales, Linda L. McCreary, Lisa Theus, Maria del Carmen Gutierrez Agudelo, Adriana da Silva Felix, Jacqueline Molina de Uriza, Nathaly Rozo Gutierrez

**Affiliations:** 1 PhD, Regional Advisor on Nursing and Allied Health Personnel, Pan American Health Organization/World Health Organization (PAHO/WHO), Washington, DC, United States of America.; 2 PhD, Professor Emeritus, School of Nursing, University of Alabama at Birmingham, Birmingham, AL, United States of America.; 3 MSc, International Consultant, Pan American Health Organization/World Health Organization (PAHO/WHO), Washington, DC, United States of America.; 4 PhD, President, Asociación Latinoamericana de Escuelas y Facultades de Enfermería (ALADEFE), Ciudad de México, DF, Mexico.; 5 MEd, Adjunct Professor, Escuela Nacional de Enfermería y Obstetricia, Universidad Nacional Autónoma de México, Ciudad de México, DF, Mexico. Director, PAHO/WHO Collaborating Centre for the Development of Professional Nursing, Ciudad de México, México.; 6 PhD, Associate Professor, College of Nursing, University of Illinois at Chicago, Chicago, IL, United States of America. Co-Director, PAHO/WHO Collaborating Centre for International Nursing Development in Primary Health Care, Chicago, IL, United States of America.; 7 MPH, Program Coordinator, PAHO/WHO Collaborating Centre for International Nursing, School of Nursing, University of Alabama at Birmingham, Birmingham, AL, United States of America.; 8 MEd, Associate Professor, Facultad de Enfermería y Rehabilitación, Universidad de la Sabana, Chía, Colombia. Executive Director, Asociación Colombiana de Facultades de Enfermería (ACOFAEN), PAHO/WHO Collaborating Centre for the Development of Innovative Methodologies in the Teaching-Learning in Primary Health Care, Bogotá, Colombia.; 9 PhD, Volunteer (2014), Pan American Health Organization/World Health Organization (PAHO/WHO), Washington, DC, United States of America.; 10 MHA, Project Administrator, Asociación Colombiana de Facultades de Enfermería (ACOFAEN), PAHO/WHO Collaborating Centre for the Development of Innovative Methodologies in the Teaching-Learning in Primary Health Care, Bogotá, Colombia.; 11 MPH, Project Coordinator, Asociación Colombiana de Facultades de Enfermería (ACOFAEN), PAHO/WHO Collaborating Centre for the Development of Innovative Methodologies in the Teaching-Learning in Primary Health Care, Bogotá, Colombia.

**Keywords:** Nursing, Nursing Education, Primary Health Care, Universal Coverage, Universal Access to Health Care Services, Nursing Education Research.

## Abstract

**Objective:**

to assess the situation of nursing education and to analyze the extent to which baccalaureate level nursing education programs in Latin America and the Caribbean are preparing graduates to contribute to the achievement of Universal Health.

**Method:**

quantitative, descriptive/exploratory, cross-sectional study carried out in 25 countries.

**Results:**

a total of 246 nursing schools participated in the study. Faculty with doctoral level degrees totaled 31.3%, without Brazil this is reduced to 8.3%. The ratio of clinical experiences in primary health care services to hospital-based services was 0.63, indicating that students receive more clinical experiences in hospital settings. The results suggested a need for improvement in internet access; information technology; accessibility for the disabled; program, faculty and student evaluation; and teaching/learning methods.

**Conclusion:**

there is heterogeneity in nursing education in Latin America and the Caribbean. The nursing curricula generally includes the principles and values of Universal Health and primary health care, as well as those principles underpinning transformative education modalities such as critical and complex thinking development, problem-solving, evidence-based clinical decision-making, and lifelong learning. However, there is a need to promote a paradigm shift in nursing education to include more training in primary health care.

## Introduction

In the Region of the Americas, many people are unable to access comprehensive health services to achieve a healthy life, prevent disease, and receive primary health care (PHC) in a timely manner. The Pan American Health Organization/World Health Organization (PAHO/WHO) proposes the Strategy for Universal Access to Health and Universal Health Coverage to improve health outcomes and other basic objectives of health systems based on the right of each person to receive the best standard of health, without exposing people to financial difficulties^1^.

Universal Access to Health is defined as the absence of geographical, economic, socio-cultural, organizational, or gender barriers and is achieved through the progressive elimination of barriers that prevent all people from using comprehensive health services, determined at the national level, and in an equitable manner. Universal Health Coverage is defined as the capacity of the health system to respond to the needs of the population, which includes the availability of infrastructure, human resources, health technologies (including medicines), and financing. For the purpose of this study, Universal Access to Health and Universal Health Coverage are jointly called Universal Health^1^.

Nurses and midwives make up the largest part of the health workforce. However, there is great variation in the levels of initial education for nursing between countries of the Region of the Americas, as well as worldwide^2^. 

Education is vital to train leaders in nursing and other health professions to create new mechanisms to achieve Universal Health. Nursing students must learn about the principles of social determinants of health, and adhere to the code of ethics and standards of the profession. The quality of education for 21^st^ century health professionals requires adequate infrastructure, partnerships, and curriculum design^2^. 

The education of health professionals for the 21st century should also be oriented towards the principles of transformative and interprofessional education^3^ ^-^ ^5^. The principles of transformative education include: (a) promotion of critical thinking; (b) promotion of professional skills development to work in teams; (c) creative adaptation of global resources to address local priorities; (d) integration of education and health systems; (e) networking and partnerships, and (f) sharing of educational resources and global innovations^3^. Interprofessional education (IPE) is one strategy to achieve transformative education and occurs when "two or more professions learn about, from and with each other to enable effective collaboration and improve health outcomes"^4^. Interprofessional education promotes teamwork and best use of valuable healthcare resources, empowering nurses to practice within the full extent of their education and training.

 The World Health Organization^6^ recommends that education institutions, to respond to transformative educational needs, adapt their institutional set-up and modalities of instruction in alignment with interprofessional education and collaborative practice. Realizing this agenda depends on giving priority to faculty development regarding facilitation of IPE to enable interprofessional learning^7^. 

In September of 2016, the High-Level Commission on Health Employment and Economic Growth^8^, established by the United Nations in collaboration with WHO and other agencies, proposed 10 recommendations to transform the health workforce for the achievement of Sustainable Development Goals. Recommendation three is related to the scale up of transformative, high-quality education and lifelong learning so that all health workers have skills that match the health needs of the population, while working to their full potential.

Nurses are essential members of the health workforce, and thus it is critical to ensure that nursing education prepares students to respond to needs of health systems and to work collaboratively in interprofessional teams^6^. Interprofessional education has the potential to transform nursing education given that it promotes the development of collaborative practice attitudes, knowledge, skills, and behaviors^7^. Ensuring that nursing educational programs adhere to principles of transformative and interprofessional education will enhance the performance and productivity of qualified health professionals and result in improved care delivery^8^. 

This study was conducted to assess the situation of nursing education in the Region of the Americas and to identify the extent to which baccalaureate level nursing education programs in Latin America and the Caribbean (LAC) are preparing graduates to contribute to the achievement of Universal Health in the region.

## Methods

The proposal for the study was submitted to the Pan American Health Organization Ethics Review Committee (PAHOERC). The committee determined that the project was exempt from PAHOERC's review because the proposal did not constitute research with human subjects and therefore did not need ethics review. 

The target population for this quantitative, descriptive/exploratory, cross-sectional study included all nursing schools with bachelor's level nursing programs in Latin America and Caribbean countries.

Lists of the schools and faculties of nursing and their contact information were provided by several sources: PAHO/WHO; the Regional Observatory of Human Resources for Health; the International Nursing Networks; the Latin America Association of Nursing Schools (ALADEFE); contacts from the Ministries of Health; national associations of nursing schools; and nursing associations and organizations in each country. EnfAmericas social networks were also used for divulgation purposes. 

Schools were contacted by PAHO/WHO through an e-mail invitation to participate. The e-mail included a description of the study proposal and objectives, link, instructions and deadline (four weeks) for completing the instrument online. In Mexico, the schools of nursing were also contacted by national nursing associations, the National Federation of Associations of Colleges and Schools of Nursing (FEMAFEE) and the Secretary of Health. Data were collected from May to September of 2016; after that date, the online survey system was closed and no other survey data were received. 

The survey instrument was developed following an exhaustive review of the literature and was based on the Donabedian model, to assess the extent to which the Structure, Process, and Outcomes of the nursing education programs prepare students to contribute to Universal Health. Donabedian developed and introduced a model for evaluating health care quality based on concepts of structure, process, and outcomes^9^. 

*Structure* focuses on the adequacy of the facilities and equipment, the suitability of the personnel and their organization, the policies and norms, administration of the organization, and communication, among others. The items related to structure in the instrument assessed the numbers of students and faculty, school policies or guidelines, classrooms, and laboratories. *Process* focuses on actions carried out to achieve the objectives. The items related to process assessed curriculum and clinical practicum experiences for the students. *Outcomes* or results focus on concrete and precise measurements of the effectiveness to the actions planned and executed that demonstrate the fulfillment of the functions and the purposes established by the organization. The items related to outcomes for this project focused on the place of employment of the graduates, and whether they were integrating competencies related to Universal Health in their work.

The first section of the instrument focused on *Structure* and included sub-sections related to: 1.1 General Information about the school; 1.2 Mission, Philosophy, and Objectives; 1.3 Resources, Infrastructure, and Relationships with the Community and External Groups; and 1.4 Policies. Sub-section 1.1 included general information questions about the context of the school: country, contact information, type of nursing education programs, numbers of students and faculty, educational preparation of faculty and number of hours of students' clinical experiences (community/primary health care vs hospital-based).

The second section on *Process* included sub-sections on: 2.1 General Professional Competencies; 2.2 Curriculum Model and Teaching/Learning Strategies; 2.3 Clinical Experiences; 2.4 Nursing Program Evaluation; and 2.5 Student Evaluation. The third section, 3.1, focused on *Outcomes* and included questions designed to evaluate whether graduates are contributing to Universal Health. 

The initial instrument included 122 items. Three assessments of content validity of the instrument were conducted and the instrument was revised by the project team before the final version was sent to nursing schools in the region. The final instrument included 68 items: 16 items related to *Structure*, 46 items related to *Process*, and 6 items related to *Outcome*s. Participants (representatives designated by deans or directors of the participating nursing schools) responded on a 5-point Likert scale indicating the extent to which they agreed that the statements in each item described their school or program (1=strongly disagree; 2=disagree; 3=neither agree nor disagree; 4=agree; 5=strongly agree). A "Does not apply" response option was also included for each item. The final version of the instrument can be obtained from the authors.

Initially the instrument was written in English and reviewed by an English native speaker. This version was then translated to Spanish and Portuguese, and reviewed by native speakers of both languages.

A link to the final version of the instrument, in the three languages separately, was made available to participants through SurveyMonkey^(r)^, an online survey software and questionnaire tool. Participants received and completed the instrument in their country's official language, with exception to Haiti that responded the survey in English. 

Data from each language version of the SurveyMonkey^(r)^ instrument were downloaded into Excel files, which were combined and then imported into the statistical analysis program SPSS Version 24. Data were analyzed using descriptive statistics including frequency distributions and analyses of mean, standard deviation, and medians for the ordinal scale items. Analyses were completed with the whole sample as well as sub-samples of responses by region, to allow identification of differences across regions of Latin America and the Caribbean.

## Results

According to the data obtained in 2016 by PAHO/WHO, a total of 1283 schools of nursing that have baccalaureate nursing programs were identified in Latin America and the Caribbean. The distribution of these schools by country is as follows: Antigua and Barbuda (1), Argentina (56), Aruba (1), Bahamas (3), Barbados (1), Belize (1), Bolivia (12), Brazil (796), British Virgin Islands (1), Cayman Islands (1), Chile (41), Colombia (46), Costa Rica (8), Cuba (5), Dominica (1), Dominican Republic (12), Ecuador (22), El Salvador (9), Grenada (2), Guatemala (6), Guyana (4), Haiti (1), Honduras (2), Jamaica (14), Mexico (135), Montserrat (1), Nicaragua (10), Panama (6), Paraguay (11), Peru (39), Puerto Rico (14), Saint Kitts and Nevis (2), Saint Lucia (2), Saint Vincent and the Grenadines (1), Suriname (3), Trinidad and Tobago (3), Uruguay (3) and Venezuela (7). No schools of nursing were identified in Anguilla, Bermuda, French Guyana, Guadalupe, Martinique, Netherland Antilles, Turks and Caicos.

Contact information was available for 1100 nursing schools (86% of the total), and each of these schools was invited to take part in the study. The final sample included 246 nursing schools (22% response rate) from 25 countries that responded to the survey after frequent contacts and reminders by phone and emails. Table 1 illustrates the response rate by country.

**Table 1:** Response Rate by Country, Latin American and Caribbean countries, 2016

Sub-Samples	Country	Number of Schools Contacted by email by PAHO/WHO*	Number of Schools Responding	Response Rate
Brazil	Brazil	796	91	11%
Mexico	Mexico	28	59**^†^**	-
Andean Area and Southern Cone	Argentina	50	15	30%
	Bolivia	8	4	50%
	Chile	22	12	55%
	Colombia	43	20	47%
	Ecuador	22	3	14%
	Paraguay	11	3	27%
	Peru	14	7	50%
	Uruguay	3	3	100%
	Venezuela	6	2	33%
Central America and Latin Caribbean	Costa Rica	8	4	50%
	Cuba	5	2	40%
	Dominican Republic	12	1	8%
	El Salvador	9	5	56%
	Guatemala	6	1	17%
	Honduras	2	1	50%
	Nicaragua	10	4	40%
	Panama	4	1	25%
	Puerto Rico	9	2	22%
Non-Latin Caribbean	Antigua and Barbuda	1	0	0%
	Bahamas	2	0	0%
	Barbados	1	1	100%
	Belize**^‡^**	1	1	100%
	Cayman Islands	1	0	0%
	Grenada	2	0	0%
	Guyana	4	0	0%
	Haiti**^‡^**	1	1	100%
	Jamaica	14	2	14%
	Montserrat	1	0	0%
	Suriname	1	1	100%
	Trinidad and Tobago	3	1	33%
Total		1100	246	22%

* PAHO/WHO - Pan American Health Organization/World Health Organization.

† More schools were contacted by national organizations in Mexico.

‡ Belize and Haiti were included in the sub-sample Non-Latin Caribbean due to the language in which the survey was answered

Data were first analyzed by all responses, and then by sub-samples. The five sub-samples analyzed were: 1. Brazil, 2. Mexico, 3. Andean Area and Southern Cone (except Brazil), 4. Central America and Latin Caribbean, and 5. Non-Latin Caribbean. The rationale for selecting these sub-samples related to the primary language spoken in the countries, and the geographic location. Data from Brazil and Mexico were analyzed separately because they had the largest number of nursing schools and the team wanted to examine characteristics of schools in these countries separately from countries with significantly fewer schools.

Table 2 provides information about the total number of full-time and part-time faculty members in the responding schools by their highest level of education. Of the 5,338 full-time nursing faculty employed in the 246 schools, 15.0% have bachelor's degrees, 12.1% have specialty degrees, 33.6% have master's degrees and 39.2% have doctoral degrees as their highest levels of education. Of the 1,951 part-time faculty, 28.5% hold bachelor's degrees, 21.5% have specialty degrees and 40.3% have master's degrees, compared with only 9.7% holding doctoral degrees.

**Table 2:** Number and Percent of Full-Time and Part-Time Professors Teaching in Baccalaureate Level Nursing Programs by the Highest Educational Level for the Total Sample and by Sub-Samples, Latin American and Caribbean countries, 2016

Highest Level of Education	Brazil N (%)			Mexico N (%)			Andean Area and Southern Cone N (%)			Central America and Latin Caribbean N (%)			Non-Latin Caribbean N (%)			Total N (%)	
	FT*	PT**^†^**		FT*	PT**^†^**		FT*	PT**^†^**		FT*	PT**^†^**		FT*	PT**^†^**		FT*	PT**^†^**
Bachelor	93 (3)	51 (8)		144 (29)	58 (45)		423 (30)	338 (35)		116 (29)	49 (35)		26 (39)	2 (5)		802 (15)	556 (28)
Specialty	237 (30)	99 (16)		47 (9)	20 (15)		282 (20)	237 (25)		72 (18)	49 (35)		6 (9)	4 (10)		644 (12)	420 (22)
Master	792 (27)	338 (53)		225 (45)	48 (37)		569 (40)	297 (31)		184 (46)	41 (29)		28 (42)	25 (66)		1798 (34)	786 (40)
Doctoral	1830 (62)	144 (23)		77 (16)	4 (3)		153 (11)	32 (3)		27 (8)	2 (1)		7 (10)	7 (18)		2094 (39)	189 (10)
Total (100%)	2952	633		493	130		1427	954		399	141		67	38		5338	1951

* Full time professors; †Part time professors

Reporting by regional sub-samples, Brazil has the highest percentage of faculty (full and part-time professors) with doctoral degrees (55.1%); the Non-Latin Caribbean (13.3%) and the Central America and Latin Caribbean (5.4%) nursing schools report the lowest percentage of faculty with doctoral degrees. A possible explanation for this finding is that Brazil has a much larger number of nursing graduate programs (master and doctoral degree), making higher degrees more accessible to nurses there, compared to other countries. If Brazil is excluded from the sample, the overall percentage of doctoral level faculty in the responding schools drops from 31.3% to 8.3%. It is important to note that Non-Latin Caribbean (37.3%) and the Central America and Latin Caribbean nursing schools (30.6%) report the largest percentages of faculty with a bachelor's degree as their highest level of education.

Table 3 presents the summary data regarding the number of Baccalaureate nursing students in each program for the total sample and each sub-sample. Based on the standard deviation of the data presented, Central America and Latin Caribbean is the sub-sample with the most variation in the number of nursing students per program: their number of reported students per baccalaureate program ranges from 30 to 4,332.

**Table 3:** Descriptive Statistics of the Number of Nursing Students in Programs by Total Sample and by Sub-Samples, Latin American and Caribbean countries, 2016

Sub-Sample	Mean	Standard Deviation	Median	Minimum	Maximum	Total Number
Brazil	281.2	230.7	222.0	30	1700	25,585
Mexico	355.5	326.5	295.0	10	1756	20,974
Andean Area and Southern Cone	497.3	504.1	340.0	5	2833	34,313
Central America and Latin Caribbean	804.0	1233.5	270.0	30	4332	16,884
Non-Latin Caribbean	187.8	135.5	133.5	50	425	1,127
Total Sample	402.0	513.2	262.5	5	4332	98,883

Table 4 illustrates the ratio of hours of clinical experiences in primary health care settings to hospital-based clinical hours by total sample and by sub-sample. For the total sample, the ratio was 0.63, indicating that students receive more of their clinical experiences in hospital settings than in primary health care settings. This ratio was highest for Brazil (0.83), and lowest for Non-Latin Caribbean schools (0.26).

**Table 4:** Descriptive Statistics and Ratio of Number of Hours in Clinical Experience Locations by Total Sample and by Sub-Sample, Latin American and Caribbean countries, 2016

Sub-Sample	Hours in Primary Health Care or Community Setting					Hours in Hospital Setting				Ratio
	Mean	SD*	Median	Range		Mean	SD*	Median	Range	
Brazil	792.2	349.0	755.5	180-2040		949.7	410.6	905.0	300-2500	0.83
Mexico	421.9	397.3	336.0	12-1765		911.7	576.5	864.0	16-2210	0.46
Andean Area and Southern Cone	690.2	581.4	545.0	90-3243		1185.6	816.6	1010.5	110-4000	0.58
Central America and Latin Caribbean	377.4	306.9	315.0	8-936		696.7	506.4	600.0	16-1604	0.54
Non-Latin Caribbean	342.3	197.4	305.0	144-700		1337.7	590.1	1465.0	240-1856	0.26
Total Sample	630.0	461.6	580.0	8-3243		995.0	613.9	920.0	16-4000	0.63

* SD = Standard Deviation

Descriptive statistics (mean, standard deviation, median) were calculated for the total sample and for each of the sub-samples, for each of the 68 instrument items. The mean for 61 of the 68 items for the total sample was greater than 4.0 on a response scale of 1-5. This finding indicates that the data are strongly positively skewed, because most responses were "Agree" or "Strongly Agree." 

In order to interpret the findings, the project team first examined all items with the greatest variability, having mean scores of 4.0 or below for the total sample as well as for the sub-samples. Only nine items had mean scores for the total sample that were 4.0 or lower, and only 12 additional items had mean scores lower than 4.0 for one or more sub-samples. 

**Figure 1:**
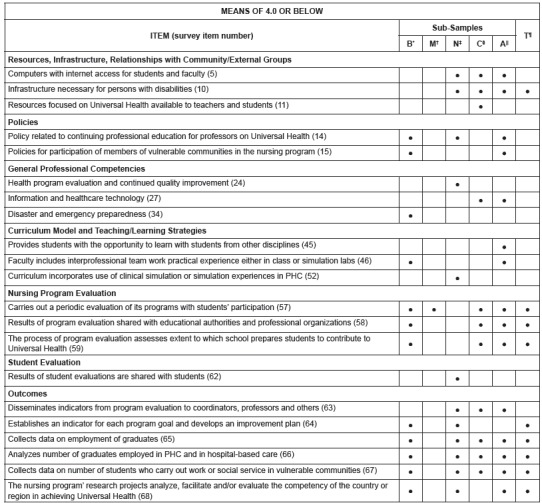
Program areas with potential for improvement by total sample and by sub-sample (means of 4.0 or below), Latin American and Caribbean countries, 2016

The items that had means of 4.0 or below are indicated in Figure 1, which also indicates the total sample or sub-sample group that had a mean below 4.0 on that item. It is important to recognize that these findings do not necessarily indicate that the responding schools are not implementing the structures, processes, and outcome evaluations reflected by the items. Perhaps some respondents did not interpret the items to be asking about what is happening at their school. However, the findings may be used to identify potential areas for ongoing program improvement.

*B= Brazil; †M= Mexico; ‡N= Non-Latin Caribbean; §C= Central America and Latin Caribbean; ||A= Andean Area and Southern Cone; ¶T= Total Sample.

**Figure 2:**
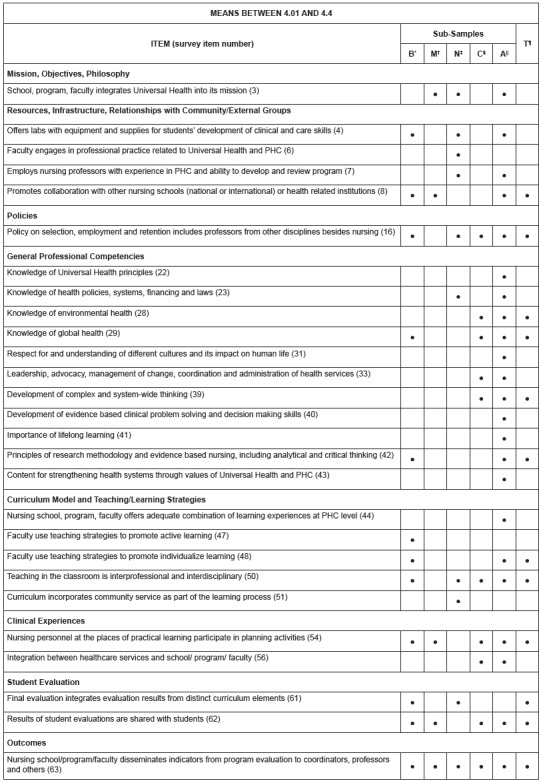
Additional program areas with potential for improvement by total sample and by sub-sample (means between 4.01 and 4.4), Latin American and Caribbean countries, 2016

Recognizing that the data were very positively skewed, we decided to expand our analysis to examine cells with means below 4.4, since we determined that 50% of the items for the total sample had mean scores that were 4.4 or lower. These items are illustrated in Figure 2. Based on this analysis, a number of the same items noted in the first analysis (means of 4.0 or below) were highlighted in other sub-samples. The following additional items were noted as having means between 4.01 and 4.4, suggesting additional potential areas for program improvement.

*B= Brazil; †M= Mexico; ‡N= Non-Latin Caribbean; §C= Central America and Latin Caribbean; ||A= Andean Area and Southern Cone; ¶T= Total Sample.

Based on the analysis of items with means below 4.0 and between 4.01 - 4.4, it appears that the major areas for improvement of nursing education programs related to *Structure* include ensuring adequate internet access for students and faculty; ensuring that programs are accessible for people with disabilities; ensuring that there are adequate resources related to Universal Health; ensuring that Universal Health is integrated into the school mission; and ensuring that there are continuing education policies for faculty related to Universal Health. Other potential areas for improvement related to structure that were noted include ensuring that members of vulnerable communities participate in the nursing program, that faculty have experience in primary health care, that there are adequate laboratory facilities for students, that the school promotes collaborations with international nursing schools and other partners, and that the school has policies related to employment of interdisciplinary faculty.

The major areas for improvement that we identified regarding *Process* were related to general professional competencies, curriculum and teaching/learning methods, and faculty and student evaluation. The general professional competency items with the lowest mean scores for the total sample included: health program evaluation, information technology, and disaster preparedness. It should be noted, however, that Brazil was the only country with means below 4.0 on the item reflecting availability of curriculum content focused on disaster preparedness. Other competencies that had mean scores of 4.4 or lower for at least three of the sub-sample groups included environmental health, global health, development of complex and system-wide thinking, and research methodology/critical thinking. 

The items with the lowest mean scores for the total sample related to *curriculum model and teaching/learning strategies* included items related to interprofessional education and providing simulation experiences in primary health care. In addition, the item related to providing individualized learning had mean scores of 4.4 or lower for three of the sub-sample groups. These results reflect a critical need for health professions educational programs in Latin America and the Caribbean to expand interprofessional education.

 The items with the greatest number of mean scores below 4.0 and between 4.1 - 4.4 were related to *nursing program evaluation, student evaluation and outcomes*. These are areas that should be high priorities of nursing schools seeking accreditation, and could be a focus for quality improvement initiatives in the region. Such initiatives include developing periodic evaluations of curricula and programs with students' participation, and sharing the results of these evaluations with educational authorities and professional organizations. 

There is also a widespread need to collect data on employment of graduates, and evaluate the number of graduates who are working with vulnerable communities. Although respondents from Mexico indicated that they collect such information from graduates, many schools in other sub-samples indicate limitations in the data they collect. Finally, there is a need to increase the research projects to analyze, facilitate and evaluate the competency of the country in achieving the Universal Health.

## Discussion

A total of 246 Schools of Nursing in 25 countries in Latin America and the Caribbean -including both public and private institutions-participated in this study. 

The ratio of hours of clinical experiences in primary health care settings to hospital-based clinical hours for the total sample (0.63) of schools surveyed indicated that students receive more of their clinical experiences in hospital settings. Brazil presented the highest ratio (0.83) and Non-Latin Caribbean the lowest (0.26).

There have been numerous recommendations regarding the importance of providing students with more clinical experience in primary health care settings, as opposed to focusing primarily on hospital settings for clinical education. In 2013, PAHO/WHO approved Resolution CD52.R13 that urges countries to promote reforms in health professions education to support primary health care-based health systems and increase the number of seats in training programs in health professions relevant to PHC. It is important to harmonize the training of health professions with the needs of the health systems based in primary health care. A paradigm shift in health sciences education should be promoted to respond to the needs of the population and the health care models with more training in primary health care services^10^.

The training of nursing students in hospital settings more than in primary health care settings is related to the labor market as well as the organization of health care models in various countries. Increasing the investment and employment in primary health care settings, with more attractive labor conditions and incentives, can strengthen and increase the desirability of positions offered to nurses in this level^1^.

Only 31.3% of faculty members from participating schools hold a doctoral degree. However, the vast majority (86%) of those faculty members with doctoral degrees in the LAC are in Brazil (1,974 faculty members). If Brazil is excluded from the sample, the percentage of faculty members with doctoral degree in LAC decreases from 31.3% to 8.3%. Most faculty members present a master's degree as their highest level of education in Mexico, the Non-Latin Caribbean, the Central America and Latin Caribbean, and the Andean Area and Southern Cone.

There are currently 51 doctoral programs in nursing in LAC. The distribution of these programs by country is as follows: Argentina (2); Colombia (2); Chile (2); Cuba (1); Jamaica (1); Mexico (2); Panama (1); Peru (1); Puerto Rico (1); Venezuela (1) and Brazil (37). PAHO/WHO is developing an action plan to increase the number of nursing doctoral programs in Latin America and the Caribbean. Nurses prepared in doctoral programs can help to fill the need for faculty positions and assume leadership roles in academics, health care services, planning and policy, thus advancing the achievement of Universal Health. 

The Future of Nursing Report^11^ recommended that nursing schools in the United States of America double the number of nurses with a doctorate by 2020, since fewer than 1 percent of nurses held a doctoral degree in nursing or a nursing related field in 2010. There is a need to increase the number of doctoral nursing programs throughout the Region of the Americas to add to the cadre of nurse faculty and researchers. Meeting the healthcare needs of the populations will require larger numbers of nurses, particularly in LAC countries, and preparing these nurses will require larger numbers of nursing faculty.

Other important findings and related recommendations from the literature are summarized below.

1) Only 64% of the schools reported that they have laboratories and equipment to help students develop care skills. Moreover, access to resources related to information and communications technologies (ICT) is still limited for both faculty and students in responding schools from Central America and Latin Caribbean, as well as Andean Area and Southern Cone countries. Access to clinical laboratories and ICT has been identified as a priority for nursing education^12^ ^-^ ^13^. Findings from this study suggest that nursing schools should work to expand laboratory and ICT resources.
2) The programs that participated in the study reported having linkages and partnerships with national or international health institutions for practical training, consistent with recommendations by the American Association of Colleges of Nursing^14^ and by Garfield^15^.
3) Between 37.8% and 42.7% of schools of nursing affirmed they have sufficient bibliographic resources to support Universal Health education for faculty and students. It is recommended that the remaining schools work to strengthen their available resources, so that Universal Health can become an integral part of the curriculum in nursing education.
4) Among the schools participating in the study, it was shown that competencies underpinning the curriculum - i.e. competencies in epidemiology, biostatistics, public health, social determinants of health, healthcare knowledge and evaluation, primary health care, and Universal Health principles - correspond to the guidelines proposed by the American Association of Colleges of Nursing^14^ and the Association of Community Health Nursing Educators^16^.
5) The high mean scores of the responses regarding curriculum reflects strong levels of knowledge regarding health system policies (i.e. laws, legislation, and funding), as well as competencies in public health, community assessment, patient safety, and public health (i.e. diseases and their management)-all these had means of 4.41 and above. However, other curriculum components that are considered to be essential by World Health Organization^5^, such as evaluation and continuous improvement of health programs, competencies on information technologies for healthcare, environmental health, global health and preparedness for emergencies and disasters, although reported fairly positively, did not reach means of 4.41.
6) Other elements that contribute to the preparedness of students to promote Universal Health - i.e. ethics; human rights; social justice; understanding of different cultures and the impact of culture on human life; leadership; advocacy; health services coordination and administration; health and therapeutic education for patients and groups in the community; knowledge of the principles of patient, family, and community centered care - should be fostered in LAC schools through learning experiences in PHC settings. Additionally, curriculum content should support the strengthening of health systems through Universal Health values and PHC, with a focus on the country's healthcare context and priorities^8^.
7) According to the World Health Organization^4^ ^-^ ^5^, education of health professionals should be oriented towards the principles of interprofessional education. Interprofessional education is an important aspect of the curriculum that supports Universal Health and should be promoted in nursing schools. However, these results show that most students do not have opportunities to learn about, from and with students from other disciplines, and interprofessional team-work is not part of the practical learning experience neither in simulation laboratories nor in the classroom. This is incongruent with recommendations from the American Association of Colleges of Nursing^14^ and the Quad Council of Public Health Nursing Organization^17^ ^)^ and may reflect a low level of faculty experience with and preparedness to engage in such teaching/learning approaches. To transform nursing education, priority needs to be given to faculty members' skill development regarding facilitation of IPE, enabling effective interprofessional learning^7^.
8) The competencies that promote complex and systemic thinking, problem solving and evidence-based care, in addition to generation of knowledge through research, only reached a mean of 4.3. In addition, the learning of principles of research methodologies only reached 4.37. These are essential elements in nurses' training that lead to development of clinical problem-solving skills and evidence-based decisions. Schools that were rated as having lower levels of these competencies should redouble their efforts to strengthen these areas.
9) Results show that, in most participating schools, the curriculum model includes community service as part of learning process, with overall mean of 4.54. The schools also offer adequate learning experiences at the primary care level, and faculty employ teaching methods that provide students with opportunities to develop individualized and active learning. These results reached a value of 4.4 and above, in accordance with studies by Frenk et al.^3^ and the World Health Organization^5^.
10) The study findings suggest that many schools are not using clinical simulation or providing clinical training experiences in PHC services, in contradiction of the recommendations proposed by the World Health Organization^5^ ^,^ ^18^. This finding suggests that more learning opportunities and clinical simulation in PHC services should be provided to the students.
11) Findings also suggest that programs could be improved by increasing the involvement of students in the planning of practical activities in primary care, as recommended by Keller et al.^19^.
12) The findings suggest the need for improvement in periodic assessment of curriculum, with students' participation, and the divulgation of its results, as recommended by Mackey et al.^20^ and the World Health Organization^18^.
13) Findings also suggest a need for improvement in development of quality monitoring, evaluation, evaluation reporting and improvement plans, as recommended by Keller et al.^19^ and the World Health Organization^18^.
14) Finally, the results regarding the number of graduates who are employed in PHC services and who currently practice in vulnerable communities show a mean below 3.97. Schools of nursing should be encouraged to promote the idea of working in primary health care upon graduation, as a way to address the health disparities in their countries. Schools also need to create information management systems to follow up with their graduates in the years following graduation and track their employment and other accomplishments.

The results of this study have many implications for future research, social communication and for improving nursing education towards Universal Health.

- In the undergraduate curricula, the practical learning experiences that support the training of human resources for health related to Universal Health should be more focused. The nursing roles and competencies that will contribute to achieving Universal Health should be made explicit in the formal curriculum, mission of the school and in the contents of the courses, as well as in the social mission of the school and the university.
- There is a need to develop and implement regional and national virtual training meetings to generate discussion toward reorienting undergraduate programs with an emphasis on Universal Health.
- It is necessary to increase the number of faculty with postgraduate degrees, by implementing the regional strategies that PAHO/WHO and other international organizations have designed for this purpose. This implies the need to commit resources to regional programs to increase the educational level of nurses and nursing faculty, particularly in Central America and Latin Caribbean, Non-Latin Caribbean and Mexico.
- There needs to be a commitment to strengthen ICT, which is essential for teaching and learning and teaching both inside and outside the classroom, including designing and implementing clinical simulation experiences focused on primary health care, as well as incorporating ICT into health education strategies.
- Leaders in simulation technology for primary health care need to be identified, who can share their expertise and contribute to training faculty in less developed countries of the region.
- Curricula need to be expanded to incorporate experiential learning for interprofessional and interdisciplinary work.
- Education programs need to take advantage of the regional infrastructure of national and international organizations and the different institutions identified as places of development and training in Universal Health, which can support the training of faculty leaders in education and services management.
- Schools should take advantage of the structure of PAHO/WHO Collaborating Centers and their associated institutions of higher education to promote the academic exchange of faculty, researchers, students and nurses, for training in PHC and Universal Health.
- Based on the successful experiences of countries in the region, schools should join together to define core competencies for Universal Health and to design a regional core curriculum for undergraduate nursing education that will produce graduates who are prepared to provide primary health care and to contribute to achieving Universal Health.
- Faculty with expertise in the primary health care arena, and those who are experienced in facilitating interprofessional education, should be hired by the schools to provide students with such important and transformative learning experiences.
- The need to increase the number of research projects related to nursing education as well as outcomes evaluation of training and nursing care.
- The need for extended access to the resources offered by the Virtual Health Library, with emphasis on the design of a specific section on Universal Health.
- The importance of addressing research priorities on Universal Health in the various national and international events in the region, to promote discussion, analysis and publication of results^21^.
- The need to gradually ensure shared decision-making for the training of health professionals by including in the regional, national and local goals the training of educational managers, deans and health service administrators in the design of policies, interventions and evaluation of results.
- The need to publish evidence of successful educational experiences and practices in the implementation of the Universal Health strategy.
- The importance of continuing studies to evaluate the role of nursing schools and to document their contribution to the health of the population over time.
- The responsibility of all health care professionals, especially educators, to urge various professional, educational and civil society groups to work in support of Universal Health initiatives.
- The need to disseminate successful experiences that have shown evidence of improvement in health with a focus on primary health care.

The instrument used for data collection was developed specifically for this study, and as such has some limitations. Although the instrument was assessed for content validity, given that this was the first time it has been used, the reliability of the instrument has not yet been established. There are also limitations associated with the sampling strategy. Sampling for this research was not representative, as it relied on contacts available to the research team. Additionally, the response rate was low, and the schools that did respond may not be reflective of the total population of nursing schools in the region. A more in-depth study by country would allow a broader picture of each country and identify particular intervention strategies specific to the country. It is also possible that some respondents may not have been fully aware of all aspects of their school's curriculum and educational approaches, and this may have resulted in an incomplete picture being reflected in their responses. Finally, the data provided by the schools were self-reported; therefore, it is possible that the data were influenced by social desirability bias. 

## Conclusions

The data obtained in the study shows the heterogeneity in nursing education in the Latin American and Caribbean region. This heterogeneity reflects the geographical, political, economic and socio-cultural disparities of each of the participating countries.

However, at the same time that diversity is observed, there are similarities in both progress and challenges. The latter represent areas of opportunity for a more comprehensive and sustained advancement of nursing education with a view to achieving Universal Health. Countries with greater development in some areas could be supportive of others in the advancement of human resources for nursing across the Region.

Findings from this study suggest that nursing curricula among the participating schools generally includes the principles and values of Universal Health and primary health care, as well as those principles underpinning transformative education modalities such as critical and complex thinking development, problem-solving, evidence-based clinical decision-making, and lifelong learning. A paradigm shift in health sciences education should be promoted to respond to the needs of the population. Even though the mean scores for most of the items were greater than 4.0, the findings can be used to identify areas for improvement to ensure that nursing graduates, as members of interprofessional health care teams, are fully prepared to make maximum contributions to Universal Health.
